# Antioxidant and Antigenotoxic Activities of the Brazilian Pine *Araucaria angustifolia* (Bert.) O. Kuntze

**DOI:** 10.3390/antiox3010024

**Published:** 2014-01-06

**Authors:** Márcia O. Souza, Cátia S. Branco, Juliane Sene, Rafaela DallAgnol, Fabiana Agostini, Sidnei Moura, Mirian Salvador

**Affiliations:** 1 Laboratory of Oxidative Stress and Antioxidants, Biotechnology Institute, University of Caxias do Sul, RS 95070560, Brazil; E-Mails: marciadenizesouza@gmail.com (M.O.S.); csbranc1@ucs.br (C.S.B); jssene@ucs.br (J.S.); rdallagnol@ucs.br (R.D.); 2 Laboratory of Biotechnology of Natural and Synthetics Products, Technology Department, Biotechnology Institute, University of Caxias do Sul, RS 95070560, Brazil; E-Mails: fagosti1@ucs.br (F.A.); smsilva11@ucs.br (S.M.)

**Keywords:** *Araucaria angustifolia*, polyphenols, antioxidant, antigenotoxicity, MRC5

## Abstract

Polyphenols are natural products with recognized potential in drug discovery and development. We aimed to evaluate the polyphenolic profile of *Araucaria angustifolia* bracts, and their ability to scavenge reactive species. The antioxidant and antigenotoxic effects of *A. angustifolia* polyphenols in MRC5 human lung fibroblast cells were also explored. The total polyphenol extract of *A. angustifolia* was determined by the Folin–Ciocalteu reagent and the chemical composition was confirmed by HPLC. Reactive oxygen species’ scavenging ability was investigated by the 2,2-diphenyl-1-picrylhydrazyl (DPPH) method and superoxide dismutase- and catalase-like activities. The protective effect of the extract in MRC5 cells was carried out by the 3-(4,5-dimethylthiazol-2-yl)-2,5-diphenyltetrazolium bromide method and the determination of oxidative lipids, protein, and DNA (alkaline and enzymatic comet assay) damage. Total phenolic content of the *A. angustifolia* extract was 1586 ± 14.53 mg gallic acid equivalents/100 g of bracts. Catechin, epicatechin, quercetin, and apigenin were the major polyphenols. The extract was able to scavenge DPPH radicals and exhibited potent superoxide dismutase and catalase-like activities. Moreover, *A. angustifolia* extract significantly protected MRC5 cells against H_2_O_2_-induced mortality and oxidative damage to lipids, proteins, and DNA. Therefore, *A. angustifolia* has potential as a source of bioactive chemical compounds.

## 1. Introduction

Plant secondary metabolites have contributed to the development of new active molecules used in therapeutics. The diversity of the plant kingdom represents an immense reservoir of structures with potential pharmacological value. *Araucaria angustifolia* (Bert.) O. Kuntze (Araucariaceae) is a native conifer of Southern Brazil, which is popularly known as “pinheiro-do-paraná” or “pinheiro-brasileiro”. It is a dioecious species, which means it features male and female specimens that have their own distinct strobilus. The female strobilus consists of seeds (the edible part of *A. angustifolia*) and bracts, which are undeveloped seeds commonly discarded into the environment.

Different parts of *A. angustifolia* are used in Brazilian folk medicine. Infusions of bark are used to treat muscle strains and varices and the syrup produced from resin is used to treat respiratory tract infections. Moreover, infusions of leaves (needles) are used to treat scrofula, fatigue, and anemia [1,2].

Despite its traditional uses, few phytochemical and pharmacological studies have been performed from *A. angustifolia*. The dead bark of the tree has a high concentration of anthocyanins and proanthocyniadins, and presents antioxidant effects on liposomes and rat microsomes [3]. *A. angustifolia* needles contain proanthocyanidins and biflavonoids such as amentoflavone, mono-*O*-methylamentoflavone, di-*O*-methylamentoflavone, ginkgetin, tri-*O*-methylamentoflavone and tetra-*O*-methylamentoflavone [4,5]. It was shown that biflavonoids exhibit minor antiherpes activity and the proanthocyanidins seem to be mainly responsible for the antiviral activity of *A. angustifolia* needles [6]. The needles can also reduce lipoperoxidation and DNA damage in liposomal membranes [4] and in calf thymus cells [5]. The resin from *A. angustifolia* is rich in lignans and phenolic compounds, such as 4-hydroxybenzaldehyde, hydroquinone and *p*-coumaric acid [7]. The seed of this tree, named *pinhão*, is rich in lectins with anti-inflammatory and antibacterial activities [8].

In our previous study [9], we found that the aqueous extract of bracts from *A. angustifolia* presents antimutagenic activity and high levels of polyphenols. These bioactive compounds can act as reducing and/or scavengers agents, minimizing the generation of reactive species (RS) [10] implicated in protein, lipid, and nucleic acid damage [9]. Therefore, polyphenols could reduce the occurrence of several diseases associated with oxidative stress, such as cancer and cardiovascular and neurological diseases [11].

The present study aimed to evaluate the polyphenolic profile of *A. angustifolia* bracts and the ability of this extract to scavenge reactive species. In addition, the effects of *A. angustifolia* in human lung fibroblast cells (MRC5) were also studied.

## 2. Experimental Section

### 2.1. Chemicals

Dulbecco’s modified eagle medium (DMEM), fetal bovine serum (FBS), trypsin-EDTA and penicillin-streptomycin were purchased from Gibco BRL (Grand Island, NY, USA). Thiobarbituric acid (TBA), trichloroacetic acid (TCA), dinitrophenylhydrazine (DNPH), 2,2-diphenyl-1-picrylhydrazyl (DPPH), hydrolyzed 1,1,3,3-tetramethoxypropane (TMP), catechin, epicatechin, quercetin, apigenin, 3-(4,5-dimethylthiazol-2-yl)-2,5-diphenyltetrazolium bromide (MTT), radio-immunoprecipitation assay (RIPA) buffer, and hydrogen peroxide (H_2_O_2_) were obtained from Sigma-Aldrich (St. Louis, MO, USA). Low-melting point agarose and normal agarose were purchased from Invitrogen (Carlsbad, CA, USA). Formamidopyrimidine DNA glycosylase (FPG, 8.000 U/mL) and endonuclease (Endo) III (10.000 U/mL) were purchased from New England BioLabs (Ipswich, MA, USA). HPLC solvents were from Mallinckrodt Baker Inc. (Phillipsburg, NJ, USA).

### 2.2. Plant Material and Preparation of the Extract

Pines of *A. angustifolia* were collected in Caxias do Sul, Rio Grande do Sul (latitude −29°10'05'', longitude −51°10'46''), Brazil, in 2011. Voucher specimens were identified by the herbarium of the University of Caxias do Sul, Rio Grande do Sul, Brazil (HUCS36536). Bracts were manually separated and mixed to obtain a pool, which was used to prepare the extract. Bracts were dried in incubator air oven at 37 °C, milled (Tecnal model Willye TE-650) and mixed with distilled water (5%, w/v). Extraction was done under reflux (100 °C) for 15 min, as described by Michelon *et al.* [9]. After cooling to 25 °C, the *A. angustifolia* extract (AAE) was filtered in Millipore equipment (pore size, 0.45 μm; catalog number SFGS 047LS, Millipore Corp., São Paulo, Brazil), lyophilized (LIOBRAS model L-101), and stored in the dark. Before each assay, the lyophilized powder was resuspended in water.

### 2.3. Determination of Total Phenolic Content and Major Compounds

Total phenolic content of the extract was measured by using the Folin–Ciocalteu colorimetric method, according to Singleton and Rossi [12], with modifications. Briefly, the lyophilized extract (5%, w/v) was mixed with 400 μL of sodium carbonate (7.5%, w/v) and 500 μL of Folin–Ciocalteu reagent. After 30 min in the dark, the absorbance was measured at 765 nm in a spectrophotometer (UV-1700 spectrophotometer, Shimadzu, Kyoto, Japan). Phenolic content of the extract was expressed as mg of gallic acid equivalents (GAE) per 100 g of bracts.

Identification and quantification of the major compounds in AAE was performed by HPLC analysis, using an HP 1100 system equipped with a UV/VIS detector (Santa Clara, CA, USA). Compound separation was performed with a 5 µm Li-Chrospher RP18 column (250 mm × 4 mm) at a flow rate of 1 mL/min. The extract was filtered on a Millipore membrane (0.45 μm) and 20 μL injected into the device. Analysis of flavonoids was performed using a binary solvent system consisting of methanol (solvent A) and a water/acetic acid mixture (100/2) (solvent B) as the mobile phase unit. Gradient conditions were: 15% solvent A and 85% B (0–30 min), 40% solvent A and 60% B (30–40 min), 75% solvent A and 25% B (40–45 min), and 85% solvent A and 15% B (45–50 min). Flavonoids were monitored by UV absorbance at 350 nm. All chromatographic procedures were performed at 25 °C. To quantify the main tannins, the extract was eluted at 1 mL/min (20 μL injection volume) using an isocratic mobile phase 90% acidic water (5% acetic acid) and 10% acidic methanol (5% acetic acid). The tannins were monitored by UV absorbance at 280 nm at 45 min. The concentrations of flavonoids (quercetin and apigenin) and tannins (catechin and epicatechin) were estimated from standard curves obtained by the analysis of various doses of standard compounds (all from Sigma-Aldrich). Results are expressed in mg/100 g bracts.

### 2.4. Radical Scavenging Activity, Superoxide Dismutase-, and Catalase-Like Activities

Antioxidant activity of the AAE was measured through the ability of the extract to donate electrons to the stable radical 2,2-diphenyl-1-picrylhydrazyl (DPPH) [13]. Briefly, the lyophilized powder was diluted to different concentrations (0.05, 0.1, 0.15, 0.2, 0.25 and 0.3 mg/mL) and added to a Tris-HCl buffer (100 mM, pH 7.0) and 250 μM DPPH dissolved in ethanol. The tubes were kept in the dark for 20 min, and absorbance was measured at 517 nm (UV-1700 spectrophotometer, Shimadzu, Kyoto, Japan). The results are represented as IC_50_ (amount of extract necessary to scavenge 50% of the DPPH radical). To evaluate the antioxidant enzyme-like activities, the lyophilized powder was diluted at a concentration of 5% (w/v). The superoxide dismutase (SOD)-*like* assay was conducted by measuring the inhibition of the rate of self-catalytic adrenochrome formation at 480 nm, in a reaction medium containing 1 mmol/L adrenaline (pH 2.0) and 50 mmol/L glycine (pH 10.2). This reaction was performed at 30 °C for 3 min [14]. Results are expressed as IC_50_ (µL from AAE needed to reduce the adrenochrome formation by 50%). The catalase (CAT)-*like* assay was performed by determining the decomposition rate of hydrogen peroxide at 240 nm [15]. Results are expressed as mmol H_2_O_2_ decomposed/minute. Catechin (0.1%, w/v) was used as a standard.

### 2.5. MRC5 Cell Culture and Treatments

MRC5 cells were cultivated under standard conditions in DMEM, supplemented with 10% heat-inactivated FBS and 1% penicillin-streptomycin. Cells were maintained in humidified atmosphere at 37 °C with 5% CO_2_. Studies were conducted when the cells reached 70%–80% confluence. AAE was added to FBS-free medium to reach the non-cytotoxic concentrations of 25 and 50 µg/mL, and incubated at 37 °C for 1 h. After this, the oxidative challenge with H_2_O_2_ (900 or 150 µM) was performed for 1 h, in the dark, in FBS-free medium. For the comet assay (alkaline and enzymatic), the treatments were performed with H_2_O_2_ at 150 μM, because 900 μM was very toxic to the DNA.

### 2.6. Cellular Viability Assay

Cell viability was measured using the MTT assay, which is based on the conversion of MTT to formazan crystals by mitochondrial dehydrogenases [16]. Cells were seeded into 96-well plates at a density of 1.0 × 10^5^ cells/mL in DMEM complete medium. After 24 h, cells were treated with AAE and H_2_O_2_ (900 μM) and then incubated at 37 °C with 5% CO_2_ for 1 h. The medium was removed and 1 mg/mL MTT dye in serum-free medium was added to the wells. Plates were incubated at 37 °C for 3 h. Subsequently, the MTT solution was removed and the obtained formazan violet product was dissolved in 100 µL dimethylsulfoxide (DMSO), stirred for 15 min and the absorbance was measured using a microplate reader (Victor-X3, multilabel counter, Perkin Elmer, Finland) at 570 nm. The absorbance of control cells was set as 100% viability and the valued of treated cells were calculated as percentage of control.

### 2.7. Determination of Oxidative Damage to Lipids and Proteins

Oxidative damage to lipids and proteins was assessed in the cells after incubation with RIPA lysis buffer for 30 min, and centrifugation at 1500× *g* at 4 °C for 5 min. The supernatant was used in both assays. Lipid damage was monitored by the formation of TBA reactive species (TBARS) during an acid-heating reaction, which has been widely adapted as a sensitive method for evaluating lipid peroxidation. Assays were performed according to Salgo and Pryor [17] with minor modifications. Briefly, 400 µL of supernatant was combined with 600 µL of 15% TCA and 0.67% TBA. The mixture was heated at 100 °C for 20 min. After cooling to room temperature, the samples were centrifuged at 1300× *g* for 10 min. The supernatant was isolated, and its absorbance was measured at 532 nm. TMP was used as a standard, and the results are expressed as nmol of TMP/mg of protein. Oxidative damage in proteins was measured by determining the carbonyl group based on the reaction with DNPH [18]. Two hundred μL of DNPH (10 mM) or 200 μL of HCl (2 M) for control were added to 50 μL of supernatants. The reaction mixture was incubated in the dark for 30 min, and vortexed every 10 min. After, 250 μL of 20% TCA were added and centrifuged at 1500× *g* for 8 min. The supernatant was discarded and the pellet was washed 3 times with ethanol-ethyl acetate (1:1) to remove free reagent. Samples were centrifuged and pellets were redissolved in 1000 μL of urea solution (8 M) at 37 °C for 15 min. Absorbance was read at 365 nm, and results are expressed as nmol DNPH/mg protein.

### 2.8. Antioxidant Activity of Superoxide Dismutase and Catalase Enzymes

After treatments, cells were incubated with lysis buffer (Tris-HCl 50 mM, pH 7.5, EDTA 5 mM, dithiothreitol (DTT) 1 mM) for 30 min, then scraped and centrifuged at 5000× *g* at 4 °C for 15 min. The supernatant was used in both assays. SOD activity was found by measuring the inhibition of self-catalytic adrenochrome formation rate at 480 nm, in a reaction medium containing 1 mmol/L adrenaline (pH 2.0) and 50 mmol/L glycine (pH 10.2). This reaction was performed at 30 °C for 3 min [14]. Results are expressed as USOD (units of enzyme activity)/mg protein. One unit is defined as the amount of enzyme that inhibits the rate of adrenochrome formation in 50%. CAT activity was measured according to the methods described by Aebi [15]. The assay determines the rate of H_2_O_2 _decomposition at 240 nm. The reaction was conducted at 30 °C for 1 min. Results are expressed as mmol H_2_O_2_/min/mg protein. All absorbances were measured in spectrophotometer model UV-1700.

### 2.9. Protein Content Determination

Cell protein concentration was determined by the Lowry method using bovine serum albumin (BSA) as a standard, according to Lowry *et al.* [19].

### 2.10. Antigenotoxicity Assay

Single cell gel electrophoresis (comet assay) was performed as described by Singh *et al.* [20]. In addition, the enzymatic comet assay was carried out to assess DNA oxidative damage. For these assays, cells were washed with ice-cold PBS, trypsinized, and resuspended in a complete medium. Then, 20 µL of cell suspension was dissolved in 0.75% low-melting point agarose and spread onto a glass microscope slide that was pre-coated with a layer of 1.5% normal melting point agarose. The slides were then incubated overnight in ice-cold lysis solution (2.5 M NaCl, 10 mM Tris, 100 mM EDTA, 1% triton X-100, and 10% DMSO, pH 10.0) to remove cellular proteins and membranes. In the enzymatic comet assay, the slides were removed from the lysing solution, washed 3 times in an enzyme buffer (40 mM HEPES, 100 mM KCl, 0.5 mM EDTA, 0.2 mg/mL BSA, pH 8.0) and incubated with 60 µL of FPG (100 m Units per gel; 45 min at 37 °C) or Endo III (100 m units per gel; 30 min at 37 °C). These enzymes recognize purine and pyrimidine oxidized bases, respectively [21]. Slides were placed on a horizontal electrophoresis unit and incubated in fresh buffer (300 mM NaOH, 1 mM EDTA, pH 13). Enough buffer was used to cover the slides for 20 min at 4 °C and to allow for DNA unwinding and the expression of alkali-labile sites. Electrophoresis was conducted for 20 min at 25 V and 300 mA. All of the above steps were performed in the dark to prevent additional DNA damage. Slides were then neutralized (0.4 M Tris, pH 7.5), stained with silver nitrate and analyzed with an optical microscope. Two hundred cells (100 cells from each of the two replicate slides) per concentration of each test were analyzed. Cells were visually scored according to tail length in five classes: (1) class 0: undamaged with no tail, (2) class 1: with tail shorter than the diameter of the head (nucleus), (3) class 2: with tail as long as 1–2 times the diameter of the head, (4) class 3: with tail more than 2 times the diameter of the head, and (5) class 4: comets with no heads. The damage index (DI) is an arbitrary score calculated for each sample, which ranges from 0 (no tail: 100 cells × 0) to 400 (with maximum migration: 100 cells × 4). In the enzymatic version of the comet assay, the damage index is the result of the subtraction of the DI of the alkaline assay of DI enzymatic assay. In both assays, the frequency (%) of the different classes of DNA damage was also evaluated.

### 2.11. Statistical Analysis

Results are expressed as mean ± standard deviation obtained from three independent experiments. Statistical significance was evaluated using one-way analysis of variance (ANOVA) with post-hoc multiple comparisons procedure (Tukey’s test). The relationships between the variables were assessed with Pearson’s product-moment correlation coefficient. Significance was accepted at *P* lower than 0.05 or 0.01. The Statistical Package for Social Sciences (SPSS, version 19.0, Armonk, NY, USA) for Windows was used for analysis.

## 3. Results and Discussion

Bioactive compounds found in plants have gained attention mainly because of their healthy benefits. Brazil is rich in biodiversity and presents six main biomes, including the Atlantic Forest biome in the south of the country. In this biome, the *A. angustifolia* is the main native species. In this study, we investigated chemical and biological effects of *A. angustifolia* bracts, a non-edible part of the plant. The results showed that AAE presents high levels of phenolic compounds (1586 ± 14.53 mg GAE/100 g of bracts). This data is in agreement with the results found for cooked seeds of *A. angustifolia* [22]. The polyphenol content of AAE was higher than that reported for other rich phenolic products, such as red wine (200.40 mg/100 mL) [23], fresh plums (366 mg/100 g) [24], and blackberries (486.53 mg/100 g) [25], showing that AAE could be a good source of polyphenols.

**Figure 1:**
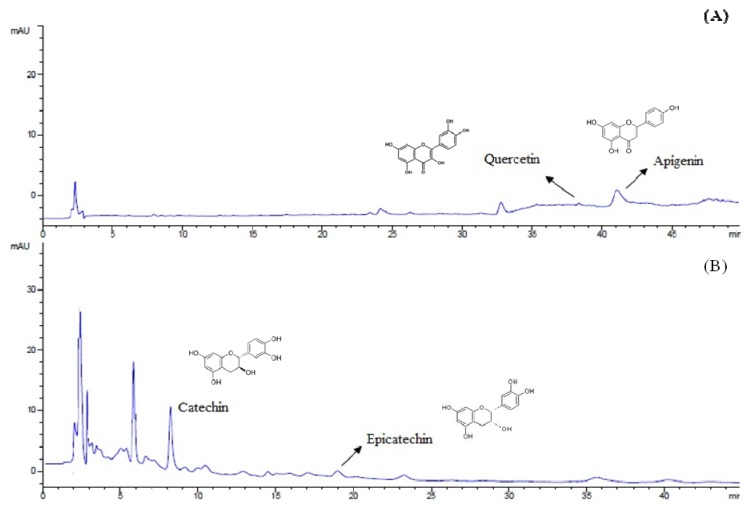
Chromatograms (HPLC) for flavonoids (**A**) at 350 nm and tannins (**B**) at 280 nm of *A. angustifolia* aqueous extract.

HPLC analysis (Figure 1) demonstrated that the major compounds of the extract were catechin (140.6 ± 2.86 mg/100 g bracts), epicatechin (41.3 ± 2.73 mg/100 g bracts), quercetin (23.2 ± 0.06 mg/100 g bracts) and apigenin (0.6 ± 0.06 mg/100 g bracts), being these last two compounds identified for the first time in *A. angustifolia* bracts.

Polyphenols are secondary metabolites characterized by the presence of two or more phenol units. According to their chemical structure, polyphenols can be divided in flavonoids and non-flavonoids [26]. Flavonoids are the most important group and present beneficial effects in cancer, cardiovascular and neurodegenerative diseases [27,28]. In general, high phenol content correlates with high antioxidant activity. Due to the presence of high amounts of polyphenols in the AAE, we evaluated the ability of the extract to scavenge the stable DPPH radical. Additionally, the capability of AAE to act as the antioxidant enzymes SOD and CAT was evaluated. SOD catalyzes the dismutation of superoxide by the production of H_2_O_2_, which can be eliminated by the action of catalase [29]. *A. angustifolia* extract showed important radical scavenging activity (IC_50_: 0.146 ± 0.14 mg/mL), measured by DPPH method. Moreover, SOD- and CAT-*like* activities were higher than those presented by standard catechin (Table 1). This indicates that AAE is able to reduce the DPPH radical and scavenge the superoxide anion and H_2_O_2_, two RS that can damage cells. Polyphenols such as catechin and quercetin have previously been reported to scavenge the superoxide radical and H_2_O_2_ [30,31]. The ability of AAE to act as an antioxidant enzyme plays an important role in maintaining the redox balance of the cells.

**Table 1:** *In vitro* antioxidant activity of the *Araucaria angustifolia* extract (AAE).

Samples	DPPH (IC_50_) ^♯^	SOD-*like* activity (IC_50_) ^§^	CAT-*like* activity (mmol H_2_O_2_/min)
AAE	0.146 ± 0.14 ^a^^,^*	5.73 ± 1.40 ^a^	225.00 ± 43.30 ^a^
Catechin	0.104 ± 0.01 ^b^	13.53 ± 0.037 ^b^	7.50 ± 0.02 ^b^

^♯^ IC_50_ (concentration of AAE (mg/mL) needed to scavenge 50% of DPPH, *i.e.*, 125 µM); ^§^ IC_50_ (µL of AAE needed to reduce 50% of the adrenochrome formation). Data are mean ± SD values. * Different letters indicate a significant difference according to analysis of variance (ANOVA) and Tukey’s post-hoc test (*p* ≤ 0.05) for each evaluated parameter.

**Figure 2:**
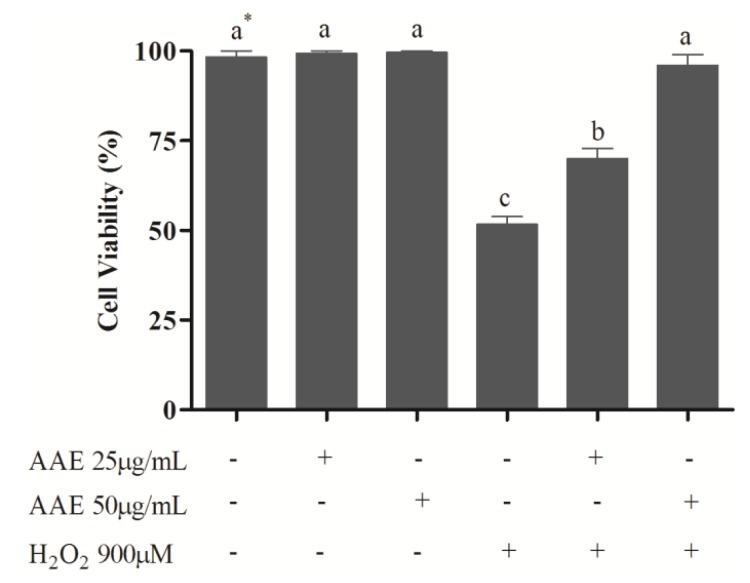
Cell viability of human lung fibroblast cells (MRC5). MRC5 cells were treated for 1 h with *Araucaria angustifolia* extract (AAE) in FBS-free medium and subsequently administered H_2_O_2_ (900 µM) for 1 h. Data are mean ± SD values. * Different letters indicate significant difference according to analysis of variance (ANOVA) and Tukey’s post-hoc test (*p* ≤ 0.05).

To study the effect of AAE in mammalian cells, MRC5 cells were treated with AAE and challenged with H_2_O_2_. The results showed that the non-cytotoxic concentrations of AAE (25 and 50 µg/mL) were able to reduce (25 µg/mL) or completely avoid (50 µg/mL) the mortality induced by H_2_O_2_ (Figure 2). In addition, AAE minimizes (25 µg/mL) or avoids (50 µg/mL) oxidative lipid and protein damage induced by H_2_O_2_. AAE also diminished (25 µg/mL) or avoided (50 µg/mL) the SOD and CAT depletion observed after H_2_O_2_ treatment (Table 2). H_2_O_2_ is generated from a variety of sources under oxidative stress and can diffuse freely in and out of cells and tissues [32] inducing cell damage. Several biomolecules can be damaged under oxidative stress and lipids are easy targets for RS. Proteins can also be damaged. The oxidation of cell membranes and amino acid side chains may lead to a loss of cell integrity and cell death [33]. To counteract RS, antioxidant enzymes, such as SOD and CAT, are the first line of defense against oxidative injury [29]. In this study, it was shown that AAE significantly protected MRC5 cells against the H_2_O_2_-induced mortality. This effect was accomplished by a reduction in oxidative damage to lipids, oxidative damage to proteins, and depletion of SOD and CAT activities induced by H_2_O_2_. These data corroborate models of similar studies, where treatment with polyphenols inhibited lipid oxidation and the decrease in SOD and CAT activities induced by H_2_O_2_ [34,35].

**Table 2:** Determination of the thiobarbituric acid reactive substances (TBARS), carbonyl protein, superoxide dismutase (SOD), and catalase (CAT) activities in the MRC5 cells pretreated with the extract of bracts of *A. angustifolia* and H_2_O_2_.

Treatments	TBARS (nmol TMP/mg of protein)	Carbonyl protein (nmol DNPH/mg of protein)	SOD (U SOD/mg of protein)	CAT (mmol H_2_O_2_/min/mg of protein)
Cell Control	0.57 ± 0.08 ^a^	1.85 ± 0.08 ^a^	19.40 ± 0.49 ^b^	15.19 ± 0.56 ^a^
H_2_O_2_ 900 µM	2.21 ± 0.06 ^c^	4.51 ± 0.15 ^c^	9.32 ± 0.13 ^d^	9.75 ± 0.08 ^c^
AAE 25 µg/mL	0.54 ± 0.01 ^a^	1.94 ± 0.10 ^a^	19.00 ± 0.34 ^b^	14.25 ± 0.65 ^a^
AAE 50 µg/mL	0.51 ± 0.15 ^a^	1.88 ± 0.06 ^a^	23.81 ± 0.01 ^a^	14.63 ± 0.06 ^a^
AAE 25 µg/mL + H_2_O_2_	0.86 ± 0.03 ^b^	3.09 ± 0.51 ^b^	13.46 ± 0.02 ^c^	12.38 ± 0.01 ^b^
AAE 50 µg/mL + H_2_O_2_	0.52 ± 0.16 ^a^	2.24 ± 0.16 ^a^	19.51 ± 0.22 ^b^	14.07 ± 0.05 ^a^

Data are mean ± SD values. Different letters indicate significant differences according to analysis of variance (ANOVA) and Tukey’s post-hoc test (*p* ≤ 0.05) in each assay.

Many methods are currently used for detecting the biological effects of DNA-damaging agents. The single cell gel electrophoresis or comet assay has been shown to be a sensitive method for investigating DNA damage. The alkaline version of this assay detects DNA strand breaks, alkali-labile sites, DNA crosslinking, and incomplete excision repair [20,21]. To verify if DNA damage is due to oxidative lesions, it is possible to use the enzymatic version of the test, performed with Endo III and FPG [21].

**Figure 3:**
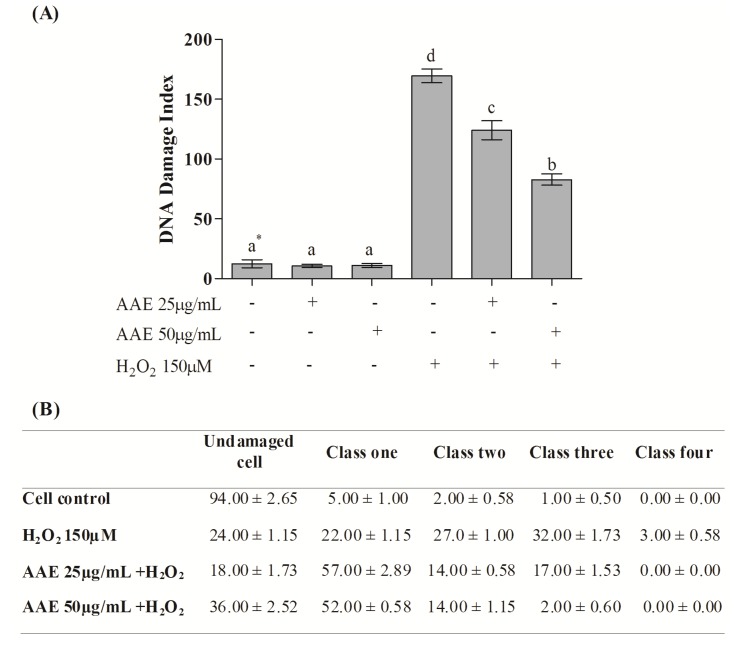
(**A**) DNA damage index by the alkaline Comet assay in MRC5 cells after treatment with AAE and exposure to H_2_O_2_. * Different letters indicate significant differences by analysis of variance (ANOVA) and Tukey’s post-hoc test (*p* ≤ 0.05). (**B**) Frequency (%) of different classes of DNA damage in control and AAE-treated groups. The cells were assessed visually and received scores from 0 (no injury) to 4 (maximally damaged), according to the size and shape of the tail. Data are mean ± SD values.

**Figure 4:**
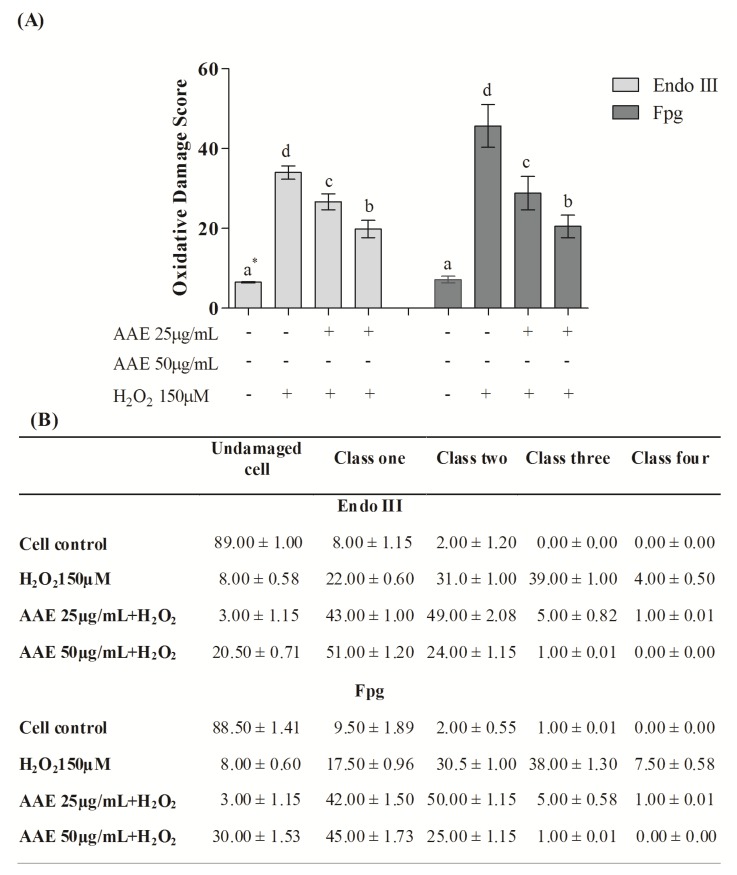
(**A**) Content of DNA damage oxidative by the Comet assay modified. * Different letters indicate significant differences by analysis of variance (ANOVA) and Tukey’s post-hoc test (*p* ≤ 0.05). (**B**) Frequency (%) of different classes of DNA damage (Comet assay). Cells were evaluated visually and were given scores of 0 (no injury) to 4 (maximally damaged) according to the size and shape of the tail. Data are mean ± SD values.

According to the alkaline comet assay, AAE alone did not cause DNA damage in MRC5 in the studied concentrations (Figure 3A). Additionally, the extract (25 and 50 µg/mL) was able to reduce DNA damage induced by H_2_O_2_ by 30% and 56%, respectively (Figure 3A). H_2_O_2_ administration induces high levels of genotoxicity, increasing the frequency of classes 2, 3, and 4 of DNA damage. This damage was reduced by AAE treatment (Figure 3B). The enzymes Endo III and FPG allowed the evaluation of DNA base oxidation. AAE reduced the oxidative damage recognized by repair protein Endo III by 21% (25 µg/mL) and 44% (50 µg/mL). AAE was also able to minimize the oxidative damage recognized by the repair FPG protein by 38% (25 µg/mL) and 60% (50 µg/mL) (Figure 4A). As observed in the alkaline comet assay, AAE treatment reduced the frequency of classes 2, 3, and 4 of DNA damage (Figure 4B). The antigenotoxic effect of AAE could be important to preventing the DNA damage associated with carcinogenesis.

Correlation analysis showed a strong negative relationship between lipid, protein, and DNA damage with SOD and CAT activities (Table 3). In addition, a strong positive correlation was found between cell viability and SOD and CAT activities, suggesting a critical detoxification by these antioxidant enzymes with a cytoprotective effect. This data suggests that SOD and CAT play an important role against lipid, protein, and DNA damage, apparently through a concerted effort that includes the dismutation of superoxide, inactivation of H_2_O_2_, and maintenance of a cellular reducing environment.

**Table 3:** Pearson correlations between cellular antioxidant enzymes activities, lipid and protein oxidative damage, DNA damage index, and cell viability assays.

Assays	SOD	CAT	TBARS	Carbonyl protein	DNA damage
TBARS	−0.866 *	−0.945 **	-	0.959 **	0.816 *
Carbonyl protein	−0.933 **	−0.992 **	0.959 **	-	0.940 **
DNA damage	−0.892 *	−0.933 **	0.816 *	0.940 **	-
Cell viability	0.943 **	0.977 **	−0.923 **	−0.985 **	−0.927 **

Statistically significant * for *p*≤0.05 and ** for *p* ≤ 0.01.

It is possible that phenolic compounds found in AAE could be responsible for the important antioxidant and antigenotoxic effects observed in this study, as already reported for this [9] and other plants [36,37,38]. However, no statistically significant correlations were found between the amount of polyphenols and the prevention of oxidative damage. This may be because we analyzed only two close concentrations (25 and 50 µg/mL) of the AAE. Assays with a wide range of concentrations as well as the evaluation of the isolated polyphenols could be helpful to identify the molecules that are responsible for the biological effects of AAE.

## 4. Conclusions

We found that AAE has an important protective effect against oxidative damage of lipids, proteins, and DNA in human lung fibroblast cells. Moreover, this extract is rich in polyphenols and is a good source of antioxidant natural compounds, mainly catechin, epicatechin, quercetin and apigenin, which present great importance in inhibiting oxidative mechanisms associate to degenerative diseases and cancer. The properties presented by the *A. angustifolia* can be used to explore new resources for pharmacological structures and/or to improve natural medicine.
